# Protein Arginine Methylation Is More Prone to Inhibition by S-Adenosylhomocysteine than DNA Methylation in Vascular Endothelial Cells

**DOI:** 10.1371/journal.pone.0055483

**Published:** 2013-02-08

**Authors:** Ruben Esse, Monica S. Rocha, Madalena Barroso, Cristina Florindo, Tom Teerlink, Robert M. Kok, Yvo M. Smulders, Isabel Rivera, Paula Leandro, Pieter Koolwijk, Rita Castro, Henk J. Blom, Isabel Tavares de Almeida

**Affiliations:** 1 Institute for Medicines and Pharmaceutical Sciences (iMed.UL), Faculty of Pharmacy, University of Lisbon, Lisbon, Portugal; 2 Department of Clinical Chemistry, Metabolic Unit, VU University Medical Center, Amsterdam, The Netherlands; 3 Institute for Cardiovascular Research ICaR-VU, VU University Medical Center, Amsterdam, The Netherlands; 4 Department of Internal Medicine, VU University Medical Center, Amsterdam, The Netherlands; 5 Department of Biochemistry and Human Biology, Faculty of Pharmacy, University of Lisbon, Lisbon, Portugal; 6 Department of Physiology, VU University Medical Center, Amsterdam, The Netherlands; Integrated Research Centre, Germany

## Abstract

Methyltransferases use S-adenosylmethionine (AdoMet) as methyl group donor, forming S-adenosylhomocysteine (AdoHcy) and methylated substrates, including DNA and proteins. AdoHcy inhibits most methyltransferases. Accumulation of intracellular AdoHcy secondary to Hcy elevation elicits global DNA hypomethylation. We aimed at determining the extent at which protein arginine methylation status is affected by accumulation of intracellular AdoHcy. AdoHcy accumulation in human umbilical vein endothelial cells was induced by inhibition of AdoHcy hydrolase by adenosine-2,3-dialdehyde (AdOx). As a measure of protein arginine methylation status, the levels of monomethylarginine (MMA) and asymmetric and symmetric dimethylated arginine residues (ADMA and SDMA, respectively) in cell protein hydrolysates were measured by HPLC. A 10% decrease was observed at a 2.5-fold increase of intracellular AdoHcy. Western blotting revealed that the translational levels of the main enzymes catalyzing protein arginine methylation, protein arginine methyl transferases (PRMTs) 1 and 5, were not affected by AdoHcy accumulation. Global DNA methylation status was evaluated by measuring 5-methylcytosine and total cytosine concentrations in DNA hydrolysates by LC-MS/MS. DNA methylation decreased by 10% only when intracellular AdoHcy concentration accumulated to 6-fold of its basal value. In conclusion, our results indicate that protein arginine methylation is more sensitive to AdoHcy accumulation than DNA methylation, pinpointing a possible new player in methylation-related pathology.

## Introduction

Cellular methylation is a crucial event in regulating gene expression and protein function. DNA methylation is an important epigenetic mechanism of gene regulation that, in differentiated cells, occurs almost exclusively by methylation of cytosine at CpG dinucleotides, forming 5-methylcytosine. DNA methylation is catalyzed by DNA methyltransferases (DNMTs). Numerous studies have addressed DNA methylation in relation to disease [1], [2].

Protein arginine methylation is a widespread post-translational modification that increases the structural diversity of proteins and modulates their function in the living cell. It is catalyzed by protein arginine methyltransferases (PRMTs), which are divided into two major classes depending on the type of methylarginine they generate [3]. Both type I and type II enzymes methylate the guanidinium nitrogen of arginine residues in proteins, forming *N^G^*-monomethylarginine (MMA). The generation of asymmetric *N^G^,N^G^*-dimethylarginine (ADMA) is catalyzed by type I enzymes, whereas type II enzymes catalyze the formation of symmetric *N^G^,N^ ´G^*-dimethylarginine (SDMA). PRMTs target a large number of distinct proteins involved in transcriptional regulation, signal transduction, RNA metabolism and DNA repair [4]. Quantification of the methylated arginine residues released upon acidic hydrolysis of proteins offers a valuable measure of protein arginine methylation status.

Methyl transfer is a single enzymatic process catalyzed by a methyltransferase. Both DNA and protein methyltransferases use S-adenosylmethionine (AdoMet) as the methyl group donor [3]. Upon the transfer of the methyl group AdoMet is converted to S-adenosylhomocysteine (AdoHcy). AdoHcy is a competitive inhibitor of most AdoMet-dependent methyltransferases because it binds to their active sites with a higher affinity than AdoMet [5]. For this reason, the ratio AdoMet/AdoHcy is regarded as an indicator of cellular methylation capacity, and a decrease in this ratio may predict a reduced cellular methylation status. AdoHcy is hydrolyzed to homocysteine (Hcy) and adenosine by AdoHcy hydrolase. This reaction is reversible, favoring AdoHcy synthesis rather than its hydrolysis. Under normal physiological conditions, Hcy is promptly metabolized by the transsulfuration and remethylation pathways and the catabolic direction of the reaction is favored, warranting a low intracellular concentration of AdoHcy [6].

Elevation of plasma levels of total homocysteine (tHcy), or hyperhomocysteinemia (HHcy), has been extensively associated with cardiovascular disease [7]. Intracellular accumulation of AdoHcy and cellular hypomethylation may underlie this relationship. Yi and coworkers [8] provided evidence that moderate elevations in plasma tHcy correlate significantly with elevations of intracellular AdoHcy levels and DNA hypomethylation in lymphocytes. This observation has been reinforced by other studies [9]–[12], highlighting the impact of one-carbon metabolism defects on global DNA methylation. Furthermore, DNA hypomethylation may be partially responsible for vascular complications associated with HHcy [13]. Thus, elevated Hcy may be regarded as a global DNA hypomethylation effector via AdoHcy accumulation.

In the past few years, several studies have cast doubt on whether elevated Hcy is indeed a risk factor for vascular disease in the general population. For instance, recent studies could not confirm the relationship between plasma tHcy level and cardiovascular morbidity and mortality if adjustment for renal function was performed, showing that plasma tHcy level depends on renal function [14], [15]. In addition, several large intervention trials to reduce tHcy level using B vitamins have been performed and have shown no benefit in terms of reducing cardiovascular risk [16]. However, HHcy may reflect disturbances in the intracellular one-carbon metabolism that are not normalized with B-vitamin treatment, as is plasma tHcy. For instance, although plasma levels of tHcy associate with plasma AdoHcy levels [8], [9], [12], it is unclear whether B vitamins decrease intracellular AdoHcy levels [17]. In a recent study, B-vitamin supplementation in an elderly population with HHcy had no effect on plasma AdoMet and AdoHcy concentrations, whereas plasma tHcy decreased [18]. If the vascular pathology associated with HHcy results from intracellular AdoHcy accumulation, the fact that B-vitamins do not decrease AdoHcy might be a clue to the lack of clinical effect of these interventions. Noteworthy, AdoHcy has been claimed as a more sensitive indicator of vascular disease than tHcy [19].

Although there is a large body of literature relating AdoMet/AdoHcy ratio to the methylation status of the cell, the extent at which protein arginine methylation is affected by AdoHcy accumulation is not known. The present study was designed to determine the effect of intracellular AdoHcy accumulation on global protein arginine methylation status, in comparison with the known AdoHcy-mediated hypomethylating effect on DNA in cultured human endothelial cells.

## Materials and Methods

### Materials

Hepes, 5-aza-2′-deoxycytidine (AZA) and adenosine-2,3-dialdehyde (AdOx) were obtained from Sigma-Aldrich (St Louis, MO, USA). L-glutamine was purchased from Biochrom-AG (Berlin, Germany). Newborn calf bovine serum and endothelial cell growth factor were from Roche (Mannheim, Germany) and collagenase, M199 basal culture medium (with Earle’s balanced salt solution and Hepes) and Hank’s balanced salt solution were from Gibco (New York, NY, USA).

### Cell Culture

Umbilical cords were obtained from the Department of Obstetrics of the Amstelland Hospital in Amstelveen, The Netherlands. The investigation conforms to the principles outlined in the Declaration of Helsinki. Endothelial cells (human umbilical vein endothelial cells, HUVEC) were isolated from human umbilical veins and cultured essentially as previously described [20]. Briefly, freshly obtained human umbilical veins were collected in a buffer solution (pH 7.3) composed of KCl 4 mmolL^−1^, NaCl 140 mmolL^−1^, D-glucose 12 mmolL^−1^, Hepes 11 mmolL^−1^, 100 UmL^−1^ penicillin and 100 mgmL^−1^ streptomycin. Within 4 to 6 days, cells were isolated by collagenase treatment and resuspended in complete M199 medium (cM199) consisting of M199 supplemented with 2 mmolL^−1^ L-glutamine, 100 UmL^−1^ penicillin, 100 mgmL^−1^ streptomycin (all Lonza, Verviers, Belgium), 10% (v/v) heat-inactivated human serum (Sanquin CLB, Amsterdam, The Netherlands), 10% (v/v) heat-inactivated new-born bovine calf serum (Gibco, Grand Island, NY, USA), 5 UmL^−1^ heparin (Leo Pharmaceutical products, Weesp, The Netherlands) and 50 µgmL^−1^ crude endothelial cell growth factor prepared from bovine brains [21]. Cells were grown at 37°C in an atmosphere of 5% CO_2_/95% air.

Cells from individual donors were grown until near confluence and passed as necessary. Before each experiment, culture medium was removed and cells were washed twice with Hank’s Balanced Salt Solution. Incubation with AdOx (2.5, 5 and 10 µmolL^−1^), a specific inhibitor of AdoHcy hydrolase, was used to stimulate accumulation of intracellular AdoHcy. AZA (5 µmolL^−1^) was employed to inhibit DNA methylation. Cells were grown during 24 h in cM199 medium with or without AdOx or AZA. The cytotoxicity of AdOx and AZA was evaluated by measuring the release of lactate dehydrogenase (LDH) into the culture medium using the Roche Cytotoxicity Detection Kit (Mannheim, Germany), in accordance with the instructions provided by the manufacturer.

Aliquots of the incubation medium were collected and stored at −20°C. Whole cell lysates were prepared by incubation in ice-cold lysis buffer (Cell Signaling Technology, Frankfurt am Main, Germany) with 1 mmolL^−1^ PMSF for 15 min and then centrifuged to remove cellular debris. Total protein was measured by the Bicinchoninic Acid Protein Assay Kit (Pierce, Rockford, IL, USA) using bovine serum albumin as the standard. Lysates were stored at −80°C until further use.

### Determination of tHcy and AdoMet and AdoHcy Levels

Total Hcy levels in culture medium before and after 24 h of incubation in unsupplemented medium or in AZA or AdOx supplemented medium were measured by the Abbott IMx fluorescence polarization immunoassay (Abbott Park, IL, USA) according to the manufacturer’s instructions. For intracellular AdoMet and AdoHcy quantification, whole cell lysates were deproteinized with equal volumes of 10% perchloric acid, centrifuged at 4°C, 16,000×*g*, for 2 min, and the obtained supernatant was analyzed by stable-isotope dilution liquid chromatography-tandem mass spectrometry (LC-MS/MS), as previously described in detail [22].

### Evaluation of Global DNA Methylation Status

DNA was obtained using a QIAamp DNA extraction kit (Qiagen, Hilden, Germany) according to the manufacturer’s protocol and quantified by measuring the absorbance at 260 nm (NanoDrop® 1000, Thermo Scientific). DNA purity was confirmed by the ratio of absorbance at 260 nm and 280 nm, which was always greater or equal to 1.8. DNA was hydrolyzed and cytosine (C) and 5-methylcytosine (mC) were analysed by LC-MS/MS, as previously described [23], [24]. In short, 1 µg of genomic DNA was hydrolyzed using 1 molL^−1^ formic acid. C and mC were separated using gradient elution reversed phase chromatography with a mobile phase containing 5 mmolL^−1^ nonafluoropentanoic acid as ion-paring reagent. Ionization was performed using positive electrospray ionization. C and mC were quantified using stable isotope dilution and the results were expressed as the percentage of methylated to total cytosine (mC/tC).

### Protein Hydrolysis

Cellular proteins were precipitated by mixing whole cell lysates with an equal volume of 20% (w/v) trichloroacetic acid. After centrifugation at 4°C, 16,000×*g*, for 10 min, the supernatants were removed and the protein pellets were washed twice with ice-cold acetone and allowed to dry at room temperature. Protein hydrolysis was carried out at 110°C with 6 molL^−1^ HCl for 16 to 24 hours. The hydrolysates were dried under a gentle stream of nitrogen gas and stored at −20°C until further analysis of ADMA and SDMA.

### Determination of Free and Protein-incorporated ADMA and SDMA

Free ADMA and SDMA levels in the incubation medium were measured by high-performance liquid chromatography with fluorescence detection, as previously described [25], [26]. Briefly, after sample cleanup by cationic solid-phase extraction (MCX 1cc cartridges, Waters Oasis, Milford, MA, USA), the analytes were derivatized with *ortho*-phthaldialdehyde reagent containing 3-mercaptopropionic acid. Chromatographic separation of the fluorescent derivatives was performed on a Chromolith Performance RP-18e column (100×4.6 mm) protected by a matching guard cartridge (10×4.6 mm) from Merck (Darmstadt, Germany). MMA was used as internal standard, since its endogenous level as free form is very low [26]. The intra-assay and inter-assay coefficients of variation were 1.4% and 2.9%, respectively, for free ADMA, and 1.4% and 3.8%, respectively, for free SDMA. For quantification of arginine (Arg), MMA, ADMA and SDMA in whole cell protein hydrolysates, homoarginine was used as internal standard, since it was of interest to quantify protein-incorporated MMA concentration. The intra-assay and inter-assay coefficients of variation of the method, considering the hydrolysis step, were, respectively, 3.6% and 4.9% for Arg, 2.8% and 6.0% for MMA, 2.6% and 4.4% for ADMA, and 5.1% and 6.6% for SDMA. The results were normalized to total Arg content of the corresponding hydrolysate.

### Real-time Reverse Transcription-PCR

Total RNA was extracted using the RNeasy Minikit (Qiagen, Valencia, CA, USA) and reverse transcribed (2 µg) into cDNA using oligo(dT) SuperScript II Reverse Transcriptase (Invitrogen, Carlsbad, CA, USA). Specific primers were designed with the Universal Probe Library Assay Design Center (Roche Applied Science, Mannheim, Germany). The sequences of the primers used were: *PRMT1*, 5′-ccaacgccaagaacaacc-3′ and 5′-tcagcgcatccggtagtc-3′; *PRMT5*, 5′-tgaattgtcgcctgagtgc-3′ and 5′-gggatgctcacaccatcat-3′; *PRMT4*, 5′-aaccacaccgacttcaagga-3′ and 5′- aaaaacgacaggatcccaga-3′; *PRMT7*, 5′-gaggacctacagggcagaaa-3′ and 5′-cagcaggctggtagtgaaga-3′; and *EIF4A2*, 5′- gtgtgaactggaccctgttg-3′ and 5′-tatttaacattcaaacttcattaagacatg-3′. *EIF4A2* served as reference gene. Amplification reaction assays were performed in triplicate and contained 1×SYBR Green PCR Mastermix (Applied Biosystems, Foster City, CA, USA) and primers at optimal concentration. Real-time PCR was performed using 40 melting and annealing/extension cycles, of 15 seconds at 95°C and 1 minute at 60°C, preceded by a 2 minute step at 50°C and a 10 minute activation step at 95°C, using the 7300 Real Time PCR System (Applied Biosystems, Foster City, CA, USA). Fluorescence emission was detected for each PCR cycle and the Ct (threshold cycle) values were determined. Normalized fold expression was calculated as difference of transcription in cells supplemented with AdOx compared to controls using the ΔΔCt method.

### Western Blotting

Western blotting was performed for analysis of PRMT 1 and PRMT5 levels, using three independent cultures. 30 cm^2^ of 80% confluent HUVEC was used for each sample. Cells were washed 3 times with ice-cold PBS, lysed with cell lysis buffer containing protease inhibitors (Sigma, St Louis, MO, USA), collected with a cell scraper, and sonicated. After centrifugation, the obtained supernatant was used for total protein determination and Western blot analysis.

Protein samples (30–40 µg) were separated on 10% SDS-polyacrylamide gels and transferred onto nitrocellulose membranes (Hybond ECLTM, Amersham, GE Healthcare, Chalfont St. Giles, UK). The membranes were incubated with anti-PRMT1 (at a 1∶500 dilution; Abcam, Cambridge, UK) or anti-PRMT5 (at a 1∶500 dilution; Millipore, Billerica, MA, USA) and anti-β-actin (at a 1∶600 dilution; Sigma, St. Louis, MO, USA) antibodies. A secondary anti-rabbit IgG HRP (Cell Signaling, Danvers, MA, USA) or anti-mouse IgG HRP (JIR, Suffolk, UK) antibody at a 1∶2,000 dilution was used. Primary antibody incubation was performed overnight at 4°C, and secondary antibody incubation was performed for 1–1.5 hours at room temperature. An ECL Plus Western Blotting Detection System (GE Healthcare, Chalfont St. Giles, UK) was used for protein detection, membranes were exposed to Amersham Hyperfilm HCl (GE Healthcare, Chalfont St. Giles, UK), and a VersaDoc scanning system (BioRad, Hercules, CA, USA) was used for densitometric analysis.

### Statistical Analysis

All experiments were performed with cells from individual donors (n ranged from 3 to 22). Results are expressed as percentage relative to cells incubated in control cM199 medium, except for free ADMA and SDMA concentrations in the incubation medium. Statistical significance was tested using Student’s paired t-test and was accepted at *P*<0.05, *versus* control.

## Results

### Effect of AdOx on Intracellular AdoHcy and AdoMet Concentrations and tHcy Production

**Figure 1:**
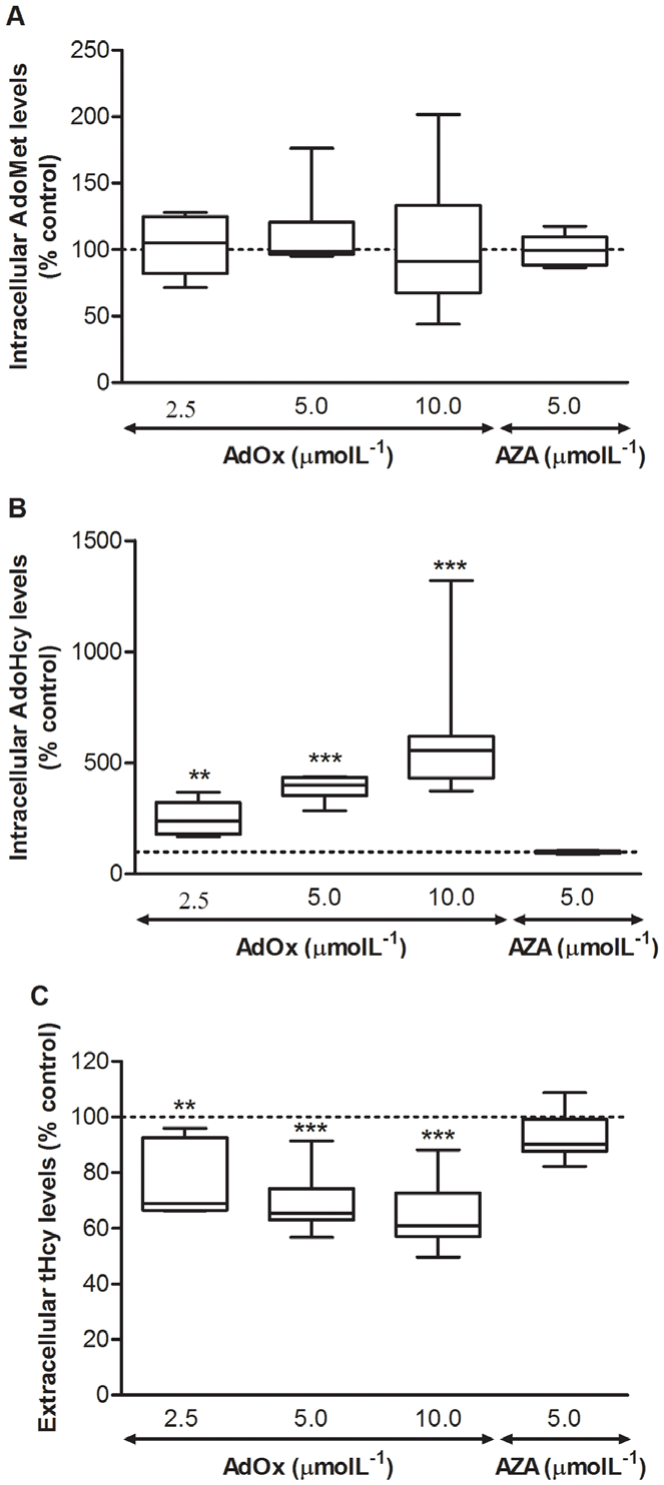
Incubation of HUVEC with AdOx, an inhibitor of AdoHcy hydrolase, induces intracellular AdoHcy accumulation and lowers tHcy production. Data represent the intracellular concentration of AdoMet (A) and AdoHcy (B) and the concentration of tHcy in the incubation medium (C) after 24 hours of incubation in the absence and presence of AdOx or AZA (see text for details), expressed as percentage of control. Data are medians, quartiles, and extreme values and represent 6 to 13 independent experiments performed with HUVEC obtained from individual donors. Statistical significance (*P*) was determined using the Student’s paired t-test; **P<0.01 and ***P<0.001, *versus* control.

To attain intracellular accumulation of AdoHcy and thereby disturb global cellular methylation processes, we used AdOx, an efficient inhibitor of AdoHcy hydrolase. As previously reported [27], AdOx elicited AdoHcy accumulation in a dose dependent manner (Figure 1B), whereas AdoMet levels did not change (Figure 1A). Furthermore, tHcy concentration in the incubation medium decreased in the presence of AdOx (Figure 1C). At the highest dose of AdOx (10 µmolL^−1^), tHcy level after 24 hours of incubation did not differ from the tHcy level present in fresh cM199 (3.0 µmolL^−1^), suggesting that, for this condition, AdoHcy hydrolase inhibition was total. Incubation with AZA, a specific inhibitor of DNA methylation, did not affect Hcy export or intracellular AdoMet and AdoHcy levels.

### Global DNA Methylation Analysis

**Figure 2:**
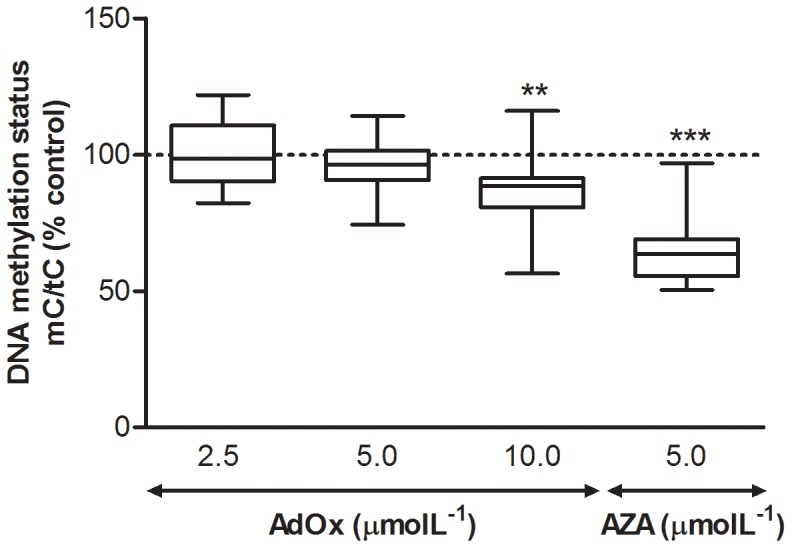
Effect of AdOx and AZA treatment on genomic DNA methylation status in HUVEC, evaluated as the ratio 5-methylcytosine/total cytosine (mC/tC). Data are medians, quartiles, and extreme values and represent 6 to 16 independent experiments performed with HUVEC obtained from different donors. Statistical significance (*P*) was determined using the Student’s paired t-test; **P<0.01 and ***P<0.001, *versus* control.

DNA methylation status, defined by the ratio of 5-methylcytosine to total cytosine in DNA hydrolysates, decreased by 10% under the highest dose of AdOx (10 µmolL^−1^), whereas no effect was observed with lower (2.5 and 5 µmolL^−1^) concentrations of the inhibitor (Figure 2). Incubation with AZA at 5 µmolL^−1^, a specific inhibitor of DNA methyltransferases (DNMTs), resulted in a 35% decrease of the 5-methylcytosine to cytosine ratio.

### Global Protein Arginine Methylation Analysis

**Figure 3:**
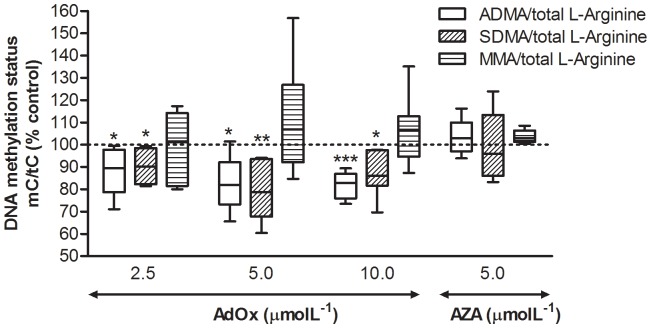
Effect of AdOx and AZA treatment on protein arginine methylation status in HUVEC, evaluated as the ratios of dimethylated arginine residues/total arginine in protein hydrolysates, after 24 hours of incubation, in the absence and presence of AdOx or AZA. Data are medians, quartiles, and extreme values and represent 5 to 7 independent experiments performed with HUVEC obtained from individual donors. Statistical significance (*P*) was determined using the Student’s paired t-test; *P<0.05, **P<0.01 and ***P<0.001, *versus* control.

The effect of intracellular AdoHcy accumulation on protein arginine methylation status was evaluated by measuring the levels of methylated arginine derivatives in the incubation medium and in cell protein hydrolysates. Basal ADMA and SDMA levels in cM199 medium were 219 nmolL^−1^ and 115 nmolL^−1^, respectively. After 24 hours of incubation, these concentrations rose to 412 nmolL^−1^ and 146 nmolL^−1^, respectively. Incubation with AdOx elicited a significant decrease in extracellular ADMA concentration, whereas that of SDMA did not change significantly (Table 1). In cell protein hydrolysates, ADMA level was about 2% of total arginine content, whereas MMA and SDMA accounted for 0.2% of total arginine content each. At all AdOx concentrations, both ADMA and SDMA decreased significantly, but not in a dose-dependent manner, while protein-incorporated MMA levels were not affected (Figure 3). AZA supplementation did not affect protein-incorporated MMA, ADMA and SDMA levels.

**Table 1:** Concentrations of ADMA and SDMA in HUVEC culture medium, after 24 hours of incubation, in the absence and presence of AdOx.

AdOx	ADMA (nmolL^−1^)	SDMA (nmolL^−1^)
(µmolL^−1^)	Mean ± SD (median [IQR])	Mean ± SD (median [IQR])
-	186±28 (189 [54])	32±15 (30 [18])
2.5	166±27 (162 [28]) **	35±13 (31 [22])
5.0	157±23 (155 [27]) **	30±18 (30 [27])
10.0	152±35 (160 [62]) **	28±18 (31 [33])

Data represent 7 independent experiments with HUVEC from individual donors. Basal levels of ADMA and SDMA in fresh medium were subtracted. Statistical significance (*P*) was determined using the Student’s paired t-test; **P<0.01, *versus* control. IQR = interquartile range.

### Effect of AdoHcy Accumulation on PRMTs’ Levels

**Figure 4:**
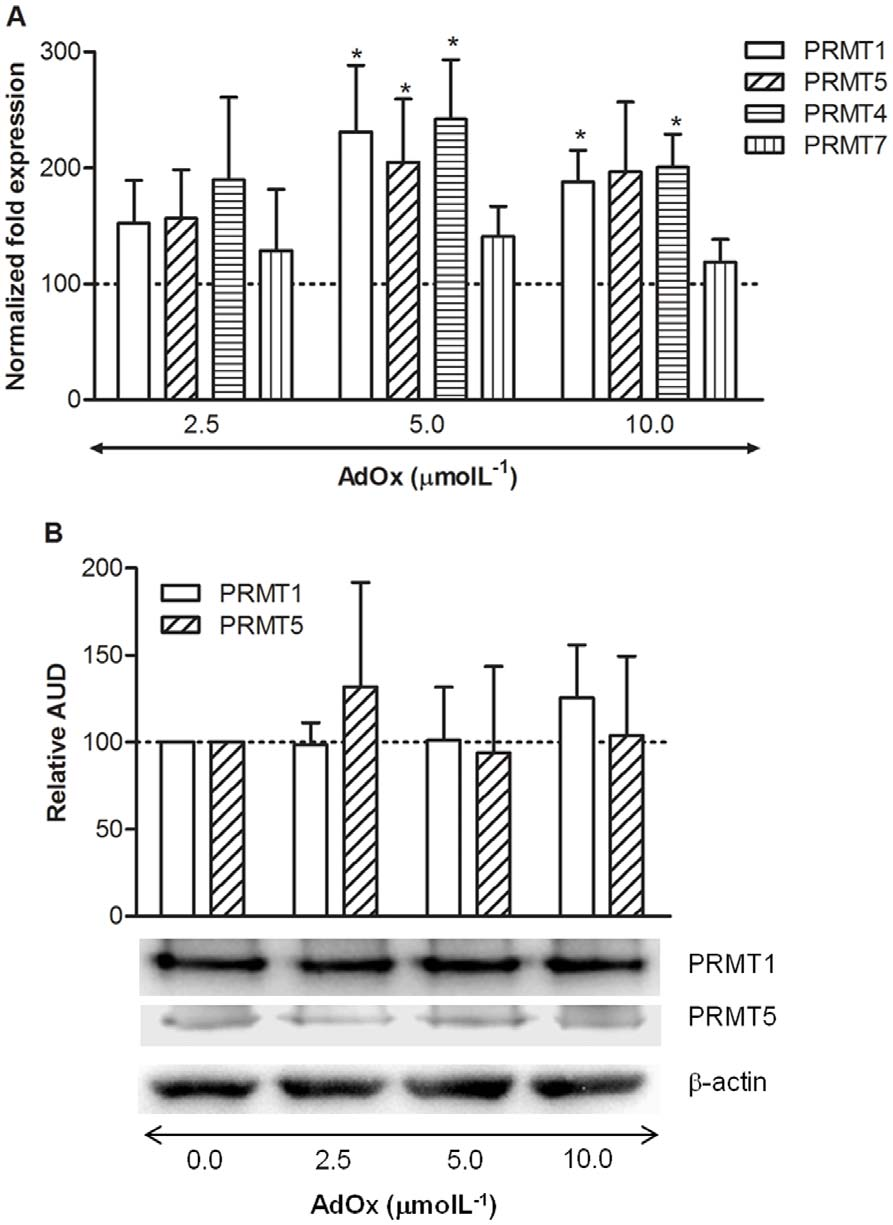
Relative PRMT1 and PRMT5 expression in HUVEC, after 24 hours of incubation, in the absence and presence of AdOx. (**A**) Real-time RT-PCR analysis was performed in triplicate for quantification of mRNA levels of *PRMT1*, *PRMT5*, *PRMT4* and *PRMT7* relative to control, using *EIF4A2* as reference gene. Data are mean±SD. (**B**) Protein levels of PRMT1 and PRMT5 determined by Western blotting, using β-actin as internal control. A representative blot is shown. Densitometry was performed on 3 blots obtained from independent experiments. Data are mean±SD. Statistical significance (*P*) was determined using the Student’s paired t-test; *P* = NS for trend and *P<0.05, *versus* control.

To investigate whether the decrease in protein arginine methylation status under AdoHcy accumulation was due to decreased levels of the enzymes responsible for this post-translational modification, we measured the transcriptional levels of PRMT1 (type I), PRMT5 (type II), PRMT4 (type I) and PRMT7 (type II). We have also determined the translational levels of PRMTs 1 and 5, the major type I and type II PRMTs, respectively, in a variety of human tissues and cells, including vascular endothelial cells [28]. There was a significant increase in the mRNA levels of PRMTs 1, 5 and 4 upon AdOx treatment (Figure 4A). The protein levels of PRMTs 1 and 5, as evaluated by Western blotting analysis, did not change (Figure 4B).

## Discussion

The present study provides evidence that accumulation of intracellular AdoHcy decreases global protein arginine methylation in cultured human endothelial cells. Importantly, we show that protein arginine methylation is more sensitive to AdoHcy accumulation than DNA methylation.

Several epidemiological surveys, as well as observations from *in vitro* and *in vivo* studies, provided evidence that AdoHcy elevation parallels HHcy [8], [9], [12], [29], [30]. Since AdoHcy is a strong methyltransferase inhibitor, a reduced cellular methylation capacity due to accumulation of AdoHcy may account for the detrimental effects associated with HHcy. In this study we aimed at investigating the direct effect of AdoHcy accumulation on protein arginine and DNA methylation status.

Incubation of cells with AdOx, a specific inhibitor of AdoHcy hydrolase, was effective in promoting build-up of intracellular AdoHcy (Figure 1B), as expected from our previously published results [27]. DNA methylation status decreased (ca. 10%) (Figure 2) but only at the highest dose of AdOx. This effect was mild in comparison with the strong decrease (ca. 35%) in DNA methylation status elicited by AZA, a specific inhibitor of DNMTs.

In order to determine the effect of AdoHcy accumulation on protein arginine methylation status, we measured the levels of free and protein-incorporated methylarginines in cell culture supernatant and intracellular protein hydrolysates, respectively. In this respect, it is important to point out that we are not mainly interested in ADMA and SDMA as indicators of cardiovascular risk factor [31] or a measure of renal function [14], [15], respectively, but rather as indicators of protein arginine methylation status. We observed that free ADMA in the culture medium decreased after 24 hours of incubation with AdOx at all doses (Table 1). In a similar study [32], Böger and coworkers reported an average 53% decrease of ADMA released to the cell culture supernatant upon treatment of ECV304 endothelial cells with AdOx at 10 µmolL^−1^ during 24 hours. In our study, ADMA release to the medium decreased only by 18% in the same conditions. This discrepancy may be due to differences on the cell lines and culture conditions used. However, both studies show that administration of AdOx reduces endothelial cell formation of ADMA, likely via AdoHcy-mediated inhibition of PRMTs. It is important to note that the levels of free ADMA in the medium do not depend exclusively on the extent of protein arginine methylation. Important events such as the turnover rate of methylated proteins, cellular export and catabolism by dimethylarginine dimethylaminohydrolase (DDAH) must be considered [33]. In order to exclude these variables and to unequivocally assess the extent at which AdoHcy accumulation affects protein arginine methylation status, we developed a method to quantify the levels of protein-incorporated methylated arginines (MMA, ADMA and SDMA). In proteins, MMA and SDMA levels were 10 times lower than those of ADMA. This result, in line with previous studies [34], [35], shows that asymmetric dimethylation is more extensive than monomethylation and symmetric dimethylation. As shown in Figure 3, accumulation of intracellular AdoHcy lowered protein-incorporated ADMA and SDMA levels in HUVEC by 10 to 20%. We conclude that both asymmetrical and symmetrical dimethylation were affected by AdoHcy accumulation. Intriguingly, the levels of SDMA in the medium did not reflect this decrease. In the study by Böger and colleagues [32], when endothelial cells were incubated in the presence of [^14^C]-CH_3_-AdoMet for 48 hours, no radioactivity could be detected in the HPLC fraction that corresponded to SDMA, suggesting that the source of SDMA released to the medium was proteins that had already been methylated before exposure to [^14^C]-CH_3_-AdoMet. Thus, for a period not greater than 48 hours, SDMA release to the medium is independent of AdoMet-dependent methylation. This observation may explain the fact that, in our study, free SDMA levels in the medium did not decrease.

To investigate whether the decrease in protein arginine methylation status under AdoHcy accumulation was due to decreased levels of the enzymes responsible for this post-translational modification, we measured the transcriptional levels of PRMTs 1, 5, 4 and 7, as well as the translational levels of PRMTs 1 and 5. Figure 4A shows that the levels of the transcripts were rather higher upon accumulation of intracellular AdoHcy, possible reflecting a compensatory mechanism secondary to PRMTs inhibition. However, at the translational levels (Figure 4B), expression of PRMT1 and PRMT5 was not affected. We conclude that the observed protein arginine hypomethylation effect was not due to reduced PRMTs levels, but probably due to inhibition of PRMTs activity. Corroborating this explanation, cell free system studies have shown that PRMT1, the main enzyme responsible for ADMA synthesis, is strongly inhibited by AdoHcy [36], [37].

In our experimental set-up, AdoHcy accumulation was accompanied by both DNA and protein hypomethylation. However, the effect on DNA methylation was only observed at the highest dose of the inhibitor (which corresponded to a 6-fold increase of intracellular AdoHcy), whereas protein arginine hypomethylation was already observed at the lowest dose of AdOx (corresponding to a 2.5-fold increase of intracellular AdoHcy). This shows that proteins are more prone to be hypomethylated by intracellular AdoHcy accumulation than genomic DNA. In fact, results from kinetic studies regarding competitive inhibition of several methyltransferases by AdoHcy support that AdoHcy is a stronger inhibitor of PRMT1 than of DNMT1 [38]–[40]. Moreover, the fact that proteins are subject to turnover, while the fraction of DNA that is susceptible to AdoHcy inhibition arises mostly from newly synthesized molecules during cell division, may contribute to this difference.

Our study has some limitations. First, only one cell type was used, which disallows us to draw a more general conclusion. Additional studies with other cell types or tissues are required to fully understand the impact of AdoHcy accumulation on protein arginine methylation in humans. Another limitation of this study concerns the approach that was adopted to stimulate AdoHcy build-up. Although incubation of cells with AdOx is commonly used, it is not known whether it impacts the physiology of the cell by mechanisms other than the inhibition of AdoHcy hydrolase. Lastly, our study does not provide a link between HHcy and protein methylation status disturbance, since we induced elevation of the Hcy precursor, AdoHcy, and not of Hcy itself. However, we think that our observations provide a valuable proof of concept and lay ground for more informative studies.

In recent years, DNA methylation status has been envisaged as a potential biomarker for several pathologies and thoroughly assayed in epidemiological studies. Many *in vivo* and *in vitro* studies have attempted to link DNA methylation status and human disease [1], [2]. However, protein arginine methylation status has been largely overlooked. In this sense, and as much as we know, our study is the first to show the extent at which AdoHcy accumulation affects protein arginine methylation status. Our results indicate that methylation of protein arginine residues is affected by intracellular accumulation of AdoHcy in a higher extension than DNA methylation. Future research is warranted to disclose the functional consequences of protein methylation disturbance in the context of Hcy-related diseases.
