# Correction: Protein Arginine Methylation Is More Prone to Inhibition by S-Adenosylhomocysteine than DNA Methylation in Vascular Endothelial Cells

**DOI:** 10.1371/annotation/40d793ec-45e1-444f-8b31-4609f1561508

**Published:** 2013-10-04

**Authors:** Ruben Esse, Monica S. Rocha, Madalena Barroso, Cristina Florindo, Tom Teerlink, Robert M. Kok, Yvo M. Smulders, Isabel Rivera, Paula Leandro, Pieter Koolwijk, Rita Castro, Henk J. Blom, Isabel Tavares de Almeida

There were errors in the Author Contributions. The correct contributions are: Conceived and designed the experiments: RE MSR RC HJB ITdA. Performed the experiments: RE MSR MB CF RMK. Analyzed the data: RE MSR TT YMS IR PL PK RC HJB ITdA. Contributed reagents/materials/analysis tools: TT PK RC HJB ITdA. Wrote the paper: RE MSR TT YMS IR PL PK RC HJB ITdA. 

